# Supplementary figures and images for: Using machine learning to investigate the relationship between domains of functioning and functional mobility in older adults
Source: PLoS One. 2021 Feb 11;16(2):e0246397. doi: 10.1371/journal.pone.0246397 (PMC7877571; doi:10.1371/journal.pone.0246397)

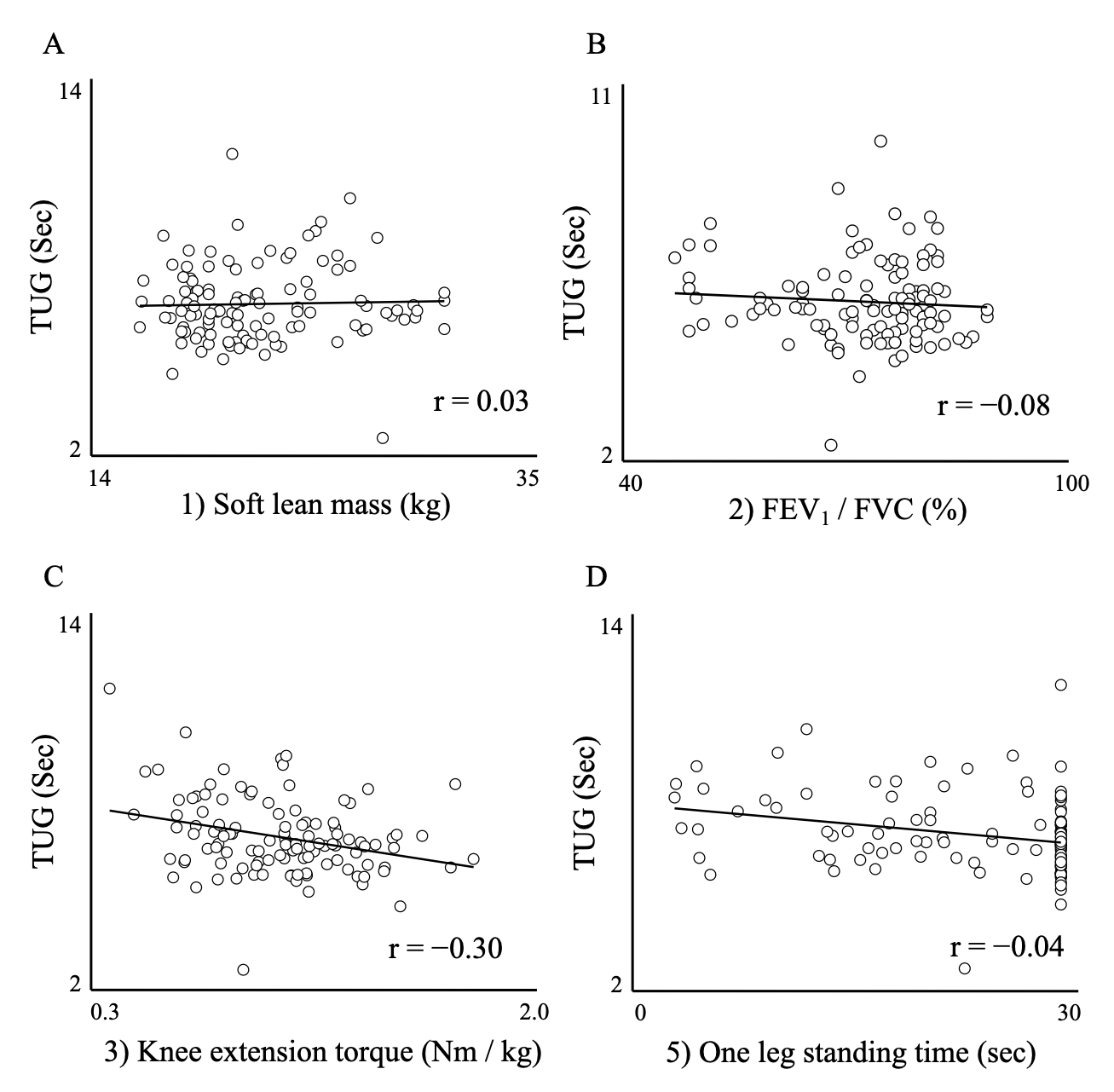

Supplement: S1 Fig — (A) soft lean mass; (B) knee extension torque; (C) FEV1 / FVC; (D) one-leg standing time. Images show correlation coefficients (r) and linear regression lines. (TIFF) [file pone.0246397.s001.tiff]

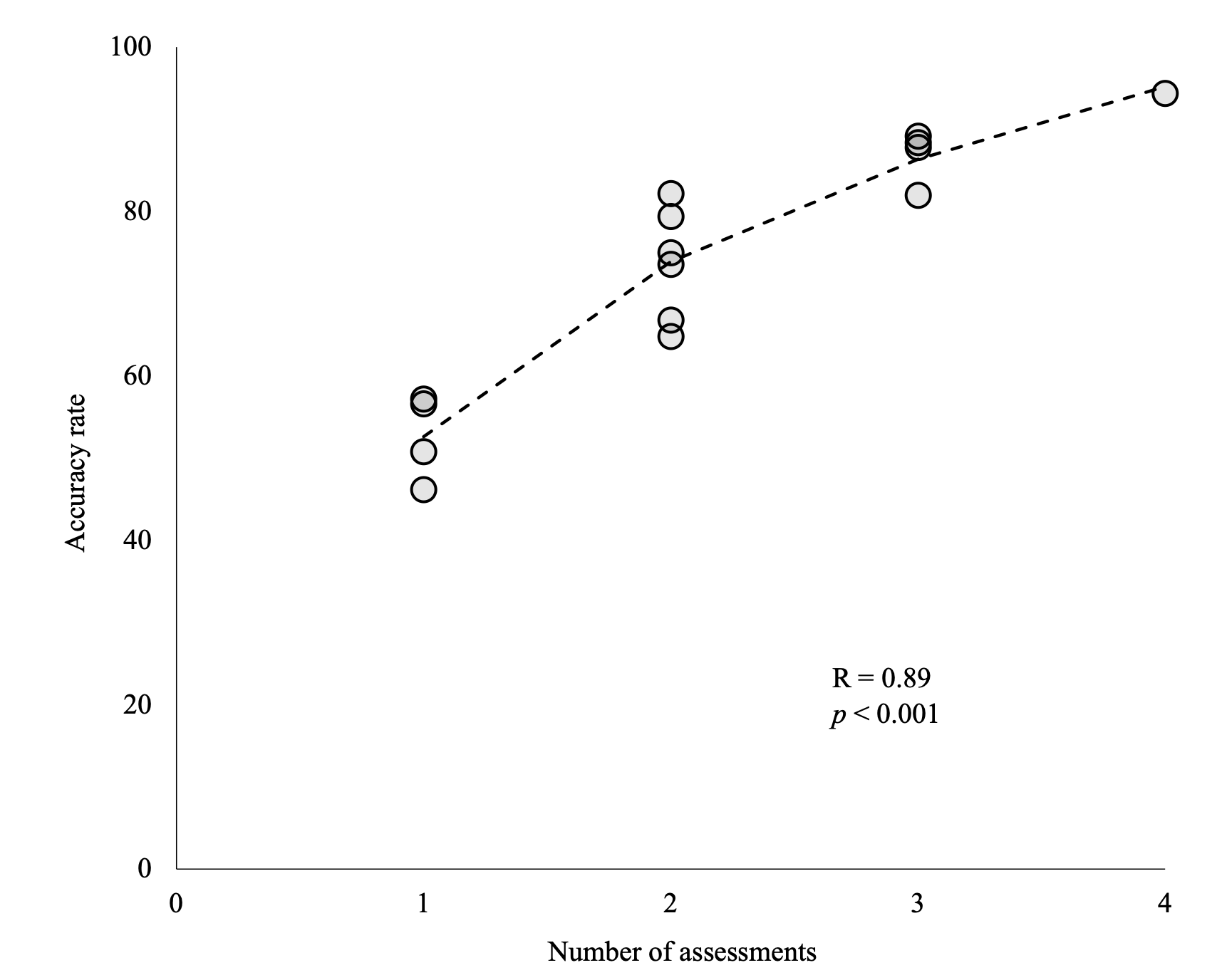

Supplement: S2 Fig — The circles show the accuracy rate of each combination of assessments and the dashed line represents the non-linear regression line. (TIFF) [file pone.0246397.s002.tiff]
