# Supplementary material for: Using machine learning to investigate the relationship between domains of functioning and functional mobility in older adults
Source: PLoS One. 2021 Feb 11;16(2):e0246397. doi: 10.1371/journal.pone.0246397 (PMC7877571; doi:10.1371/journal.pone.0246397)
Supplement: Attachment — Submitted filename: Peer-Review_12.2020.pdf [file pone.0246397.s003.pdf]

This study included 112 participants to conduct Support Vector Machine (SVM) prediction for five Timed Up and Go (TUG) rank-based groups, with the input of all 15 kinds of combinations of four assessments in each domain (body structures: soft lean mass; body functions: FEV1 / FVC, knee extension torque; activities: one-leg standing time). The results in the present study showed that knee extension torque was the most closely related to each participant's TUG rank, and made the biggest contributions to an accurate prediction.

The study is of interest and the manuscript is written rather well. However, I have several comments, possibly helping to improve the overall scientific quality of the paper:

1, The authors should improve the introduction of their study with clear aims and hypotheses. Additionally, comparing with previous studies and clarifying the reasons for method selection, what are the benefits and drawbacks of SVM? The explanation of SVM on page 4, lines 65-66 is incorrect. The kernel function is not the way to avoid overfitting.

2, The machine learning method SVM was used in this study. However, functional mobility prediction is a research area with plenty of effective methods and algorithms that can be applied to obtain a highly accurate classification. Therefore, whether other much transparent methods (for example, the methods Random Forest (RF), Artificial Neural Network (ANN) or Partial Least Square Discriminant Analysis (PLS-DA)) may achieve better prediction and interpretable results than the SVM method but not used in this study need to be carefully discussed.

3, Moreover, a clear procedure of study should be claimed in the method part.

a) how many participants are exactly involved in as the input in the model? Because in the method section, the sample size was shown as 90, but in the results section, the input sample size was 121 or 112 (the statement was confusing);
b) it was not clear how and why the sample size needs to be extended to 1000 and what the benefits are; since the present study only included a maximum of four variables.
c) how the hyper-parameters of SVM were trained, which kernel function was used in the present study? Commonly, a valid hyper-parameter can be identified by using a validation set that is split from the 90% training set, or using a cross-validation method
d) whether all data as inputs to SVM model were standardized to make sure all variables have the same unit? Since multiple-unit variables can negatively impact the SVM model.

4, The output parameters of the prediction models in Table 2 only show accuracy. However, in order to show the model is valid and not overfitting, a comprehensive evaluation with other validation matrices should be provided, such as sensitivity, specificity and AUC.

5, On page 8, line 173, the statement is incorrect, SVM is not a kernel function, it was a classification or prediction method includes a kernel function. How the SVM addresses the

multicollinearity of data in the present study (e.g., linear separately in high dimensional space) should be interpreted.

6, On page 8, lines 176-177, the expression "*SVM projection enabled us to use information that was removed when selecting variables*" is unclear. Who removes which variables? Or you mean no need for a prior variables' selection?

7, On page 9, line 190, the statement "*a larger number of explanatory variables*" is not proper because you only show four variables in the present study.

8, On page 3, line 39, repeated references; lines 49-50, e.x. should be, e.g.,
